# Exploring Anti-Fibrotic Effects of Adipose-Derived Stem Cells: Transcriptome Analysis upon Fibrotic, Inflammatory, and Hypoxic Conditioning

**DOI:** 10.3390/cells13080693

**Published:** 2024-04-17

**Authors:** Marvin L. Frommer, Benjamin J. Langridge, Alexandra Beedie, Sara Jasionowska, Laura Awad, Christopher P. Denton, David J. Abraham, Jeries Abu-Hanna, Peter E. M. Butler

**Affiliations:** 1 Charles Wolfson Centre for Reconstructive Surgery, Royal Free Hospital, London NW3 2QG, UK; 2 Department of Surgical Biotechnology, Division of Surgery & Interventional Science, University College London, London NW3 2QG, UK; 3 Department of Plastic Surgery, Royal Free Hospital, London NW3 2QG, UK; 4 Centre for Rheumatology, Department of Inflammation and Rare Diseases, Division of Medicine, University College London, London NW3 2QG, UK; 5 Division of Medical Sciences, University of Oxford, Oxford OX3 9DU, UK

**Keywords:** skin fibrosis, fibrosis, adipose-derived stem cells, RNA sequencing, transforming growth factor beta 1, interleukin 1 beta, hypoxia, fetal bovine serum

## Abstract

Autologous fat transfers show promise in treating fibrotic skin diseases, reversing scarring and stiffness, and improving quality of life. Adipose-derived stem cells (ADSCs) within these grafts are believed to be crucial for this effect, particularly their secreted factors, though the specific mechanisms remain unclear. This study investigates transcriptomic changes in ADSCs after in vitro fibrotic, inflammatory, and hypoxic conditioning. High-throughput gene expression assays were conducted on ADSCs exposed to IL1-β, TGF-β1, and hypoxia and in media with fetal bovine serum (FBS). Flow cytometry characterized the ADSCs. RNA-Seq analysis revealed distinct gene expression patterns between the conditions. FBS upregulated pathways were related to the cell cycle, replication, wound healing, and ossification. IL1-β induced immunomodulatory pathways, including granulocyte chemotaxis and cytokine production. TGF-β1 treatment upregulated wound healing and muscle tissue development pathways. Hypoxia led to the downregulation of mitochondria and cellular activity.

## 1. Introduction

Skin fibrosis shares common pathophysiological processes of fibrosis observed in other fibrotic organs with excessive production and deposition of extracellular matrix (ECM) components [1]. During wound healing, damaged epithelial and endothelial cells release factors associated with tissue damage including tumour necrosis factor (TNF) and interleukins (e.g., IL-1β). These factors initiate an inflammatory response with the influx and activation of immune cells (neutrophils, macrophages, monocytes, mast cells, etc.) [1]. Subsequently, fibroblasts become activated and acquire a myofibroblast phenotype, exhibiting the expression of contractile proteins and producing large amounts of ECM components [1,2]. After wound healing is completed, myofibroblasts quiesce and undergo apoptosis. However, during fibrosis, the stimuli (repetitive trauma, chronic inflammation, toxins) persist, and the tissue enters a cycle of ineffective or ‘frustrated’ wound healing with persistent activation of myofibroblasts depositing collagen and increasing tissue stiffness [3,4,5]. Tissue fibrosis is associated with the disruption and loss of microvessels due to pericyte loss and perivascular contraction, the occlusion of microvessels, the inability of vessels to sprout into the stiff tissue, and an increased demand for oxygen due to an influx of inflammatory and mesenchymal cells to the site of injury [3,4,5]. The resulting hypoxia leads to the accumulation of reactive oxygen species (ROS), which are primarily produced by macrophages [6]. While their antimicrobial activity is necessary for normal wound healing, they can cause damage to the tissue in the dysregulated fibrotic environment. ROS strongly activate the TGF-β/SMAD axis, leading to additional downstream collagen accumulation [7]. Transforming growth factor beta 1 (TGF-β1) is among the main activators of ECM synthesis upon fibroblast activation is [8,9]. TGF-β1 is produced primarily by immune cells and is an essential growth factor in wound healing. It can be stored latent in the ECM and activated by tissue deformation, damage, and ECM degradation [9]. Dysregulation of the TGF-β/SMAD pathway is strongly linked to the development of tissue fibrosis, and elevated levels of TGF-β1 are shared across all fibrotic conditions.

In skin fibrosis, collagen is deposited in the dermis, which ultimately leads to a loss of function. The skin reaches a state of chronic inflammation, it becomes stiff and thickened, and adnexal structures such as sweat glands and hair follicles are lost along with the underlying adipose tissue [10]. Skin fibrosis with symptoms such as microstomia in scleroderma significantly reduces the quality of life [11]. Current treatment options remain sub-optimal and have significant side-effect profiles.

Autologous fat grafting (AFG) is a commonly used reconstructive technique for correcting volumetric tissue deformities. Recent studies have observed that fat grafting not only serves as volumetric reconstruction but also enhances the quality of the surrounding tissue, reversing scarring and stiffness and ultimately improving the patient’s quality of life. These benefits have been investigated for treating a variety of fibrotic skin diseases including radiation-induced fibrosis, scleroderma, lichen sclerosis, Dupuytren’s disease, and scars in general [12,13,14,15]. Through investigating these anti-fibrotic effects, studies found that AFG led to a reduction, remodelling, and realignment of collagen fibres, increased vascularization, and a reduction in α-SMA, dermal thickness, and scar size [16]. AFG also enhanced aesthetic appearances with less decolouration and reduced painfulness [17,18]. However, the exact mechanisms by which AFG improves skin fibrosis remain poorly understood.

Although only accounting for a small percentage of the cells present in lipotransfers, adipose-derived stem cells (ADSCs) are hypothesised to be of central importance in the anti-fibrotic effects of AFG. ADSCs were discovered in lipoaspirates in 2001 [19]. They are multipotent adult stem cells with the capacity for self-renewal, long-term viability, and multilineage potential [20]. ADSCs are easier to harvest than bone marrow derived mesenchymal stem cells (BM-MSCs), with plentiful donor tissue and minimal morbidity and with significantly fewer ethical implications than embryonic stem cells; as such, they have since been investigated widely for applications in regenerative medicine. ADSCs can differentiate into various cell types of the mesenchymal lineage, including adipocytes, fibroblasts, myocytes, keratinocytes, chondrocytes, and osteoblasts, but also of other lineages such as neural cells, endothelial cells, and hepatocytes [20]. ADSCs are part of the stromal vascular fraction (SVF), which consists of a mix of cells including tissue-resident lymphatic cells, macrophages, endothelial cells, smooth muscle cells, pericytes, and preadipocytes [21].

Three mechanisms of action of ADSCs have been proposed to counteract fibrosis: regenerative (1), direct cell-to-cell-contact dependent (2), and paracrine (3). However, their paracrine signalling or secretome is considered the most dominant mechanism by which they counteract inflammation, promote wound healing, and possibly reverse fibrosis. ADSCs interact with a variety of cell types including immune cells, endothelial cells, and fibroblasts—all key drivers of fibrosis [16,17,18,22,23]. They are believed to exert their anti-fibrotic effects by the secretion of growth factors including basic fibroblast growth factor (FGF2), platelet derived growth factor (PDGF) and hepatocyte growth factor (HGF) immunomodulatory molecules (IDO, PGE2, IL10, CSF2), proangiogenic factors including vascular endothelial growth factor (VEGF), matrix remodelling enzymes, e.g., matrix metalloproteinases (MMPs), pro-apoptotic factors, and extracellular microvesicles [16,17,18,22,24]. This leads to myofibroblast apoptosis, endothelial cell growth, macrophage polarization, the suppression of lymphocyte proliferation, and natural killer cell cytotoxicity, amongst others [16,17,18,22].

This current study aims to investigate the response of ADSCs to the profibrotic and proinflammatory factors TGF-β1 and IL1-β and hypoxia at the transcriptomic level. Our approach was designed to better understand the effects of ADSCs in vivo and contribute to the development of novel anti-fibrotic therapies.

## 2. Methods

### 2.1. Donor Specifications

Human ADSCs were obtained from five donors (*n* = 5) without systemic disease. Ethical approval was obtained from the research ethics committee at the Royal Free Hospital and University College London (ethics reference: 16/LO/1603). Three donors were female (3/5 female), the average age was 53 years, and the average BMI was 23.

### 2.2. Isolation of ADSCs

Samples were obtained from excess fat during lipotransfer procedures utilising blunt abdominal liposuction. The samples were incubated at 37 °C for 30 min with collagenase type I (ThermoFisher Scientific, Waltham, MA, USA). Then, Dulbecco’s modified eagle medium (DMEM, ThermoFisher Scientific, Waltham, MA, USA) with 10% fetal bovine serum (FBS, ThermoFisher Scientific, Waltham, MA, USA) was added and the samples were filtered through a 70 µm cell strainer (VWR International, Radnor, PA, USA). Finally, the samples were centrifuged at 300× *g* for 5 min. The cell pellet was resuspended in Dulbecco’s modified eagle medium: nutrient mixture F-12 (DMEM/F12, ThermoFisher Scientific, Waltham, MA, USA) supplemented with 1% (*v*/*v*) penicillin–streptomycin (P/S, Sigma-Aldrich, Merck KGaA, Darmstadt, Germany) and 10% (*v*/*v*) FBS, transferred to a T25 flask (Corning, Corning, NY, USA), and placed in a humidified atmosphere of 5% CO_2_ at 37 °C.

### 2.3. Culture of Human ADSCs

ADSCs were maintained in culture in DMEM/F12 for two passages at 37 °C in a humidified atmosphere of 5% CO_2_. At approximately 80% confluency, the cells were replated or used for experiments. To detach the cells, the T25 flasks were washed with phosphate-buffered saline (PBS, ThermoFisher Scientific, Waltham, MA, USA) and trypsinised with TrypLE Select (ThermoFisher Scientific, Waltham, MA, USA). After centrifugation at 300× *g* for 5 min, the cell pellet was resuspended. The cells were counted and their viability was assessed using trypan blue, 0.4% (ThermoFisher Scientific, Waltham, MA, USA).

### 2.4. Flow Cytometry

ADSCs at passage 3 and prior to treatment were characterised by flow cytometry. The ADSCs were stained with anti-human antibodies for the following CD antigens (Myltenyi Biotec, Bergisch Gladbach, Germany): CD90 FITC, CD73 PE, CD105 APC, CD34 PE-Vio615, CD31 VioBlue, and CD45 APCVio770 as well as the viability dye Zombie Agua (BioLegend, San Diego, CA, USA). The markers were chosen based on recommendations from a joint statement of the International Federation for Adipose Therapeutics and Science (IFATS) and the International Society for Cellular Therapy (ISCT) [25]. Compensation was performed using the MACS Comp Bead Kit, anti-REA (Miltenyi Biotec, Bergisch Gladbach, Germany). Data acquisition was performed using a MacsQuant Analyser 10 and MacsQuant analysis software. Data analysis was performed using FlowJo^TM^ v.10.8.1 (BD Biosystems, Franklin Lakes, NJ, USA). Subsequently, cell debris, cell duplets, and dead cells were eliminated from the samples. Live cells were defined as cells with a fluorescence < 10^3^ for Zombie Agua. Then, cells that were positive for CD73^+^ and CD90^+^ and negative for CD45^−^ and CD31^−^ were obtained by sequential gating.

### 2.5. Treatment with FBS, IL-1β, TGF-β1 and Hypoxia

ADSCs were seeded in 6-well plates at a density of 100,000 cells/well and maintained in DMEM/F12 with 10% FBS for 24 h. The ADSCs were then starved in DMEM/F12 with 0.1% FBS for 24 h to induce growth arrest. After serum starvation, the ADSCs were treated under four different conditions. The control group was maintained in DMEM/F12 with 0.1% FBS and no supplements. The other groups were treated with (A) 10% FBS (Life Technologies), (B) 2 ng/mL TGF-β1 (R&D Systems, Minneapolis, USA) [26], (C) 10 ng/mL IL1-β (R&D Systems) [27], or (D) grown in a hypoxia chamber at 1% O_2_ [28]. Following 48 h of treatment, cells were collected and used for RNA isolation.

### 2.6. RNA Isolation and Bulk Sequencing

To analyse gene expression through RNA bulk sequencing, the total RNA from the cells grown in a monolayer was extracted using the RNeasy Mini Kit (Qiagen, Hilden, Germany) according to the manufacturer’s instructions. The RNA concentration and purity were assessed using a NanoDrop One Microvolume UV-Vis Spectrophotometer (ThermoFisher Scientific, Waltham, MA, USA). The RNA samples were stored at −80 °C until further use. The preparation of the RNA library and transcriptome sequencing was conducted by Novogene Co., Ltd. (Beijing, China). Quality control (QC) of the samples was performed using the 5400 Fragment Analyzer system (Agilent Technologies, Inc., Santa Clara, CA, USA). They required a minimum amount of 200 ng and 4.0 integrity for each RNA sample. All the samples passed QC. In brief, the Illumina NovaSeq 6000 Sequencing System (Illumina, Inc., San Diego, CA, USA) was used for sequencing with a read length of paired-end 150 bp and ≥20 million read pairs per sample. The genome was mapped to the Genome Reference Consortium Human Build 38 (hg38). The average raw read count per sample was 46,006,243.2 with an average sequencing error rate of 0.0224.

### 2.7. Data Analysis

Data analysis was performed in R (version 4.2.2). Principal component analysis (PCA) was performed to calculate sample distances and the effect of treatment. The results were visualised using the R package PCAtools. DESeq2 was used for differential expression analysis [29], and significantly differentially expressed genes (DEGs) were selected based on adjusted *p* values < 0.01 and a log2-fold change (log2fc) threshold = 1. The DEG heatmap was created using ggplot2. Pearson correlation was used for rows while Spearman correlation was used for columns. Z scores were computed based on normalized count data, with normalization conducted using DESeq2 counts (dds, normalized = T). Intersections and commonalities between the samples were further visualised using the R package UpSetR [30]. The EnhancedVolcano package was used to create Volcano plots. For gene set enrichment (GSEA), DEGs were ordered by their log2fc and used as input. The R package ClusterProfiler was used and the gene sets were aligned with the Gene Ontology (GO) database [31]. GSEA was performed with a *p*-value cutoff of 0.05, a gene set size of 10 to 500, 10,000 permutations, and eps set to 0. ClusterProfiler’s inherent function simplify was used to remove redundant terms at a cut-off of 0.7 and 0.55 for the FBS group. Predefined GO gene sets were then downloaded from the molecular signatures database and used to filter the DEG lists [32]. The gene sets used to filter the DEGs were “*GOCC_COLLAGEN_CONTAINING_EXTRACELLULAR_MATRIX*”, “*GOBP_WOUND_HEALING*”, “*GOBP_IMMUNE_RESPONSE*” and can be viewed at https://www.gsea-msigdb.org/ (accessed on 7 December 2023).

## 3. Results

### 3.1. ADSCs Exhibit Distinct Transcriptomic Profiles following In Vitro Treatments

Isolated human ADSCs were characterised by flow cytometry (Supplementary Figure S1). In total, 97.9% of all live cells were negative for both the hematopoietic marker CD45 and the endothelial cell marker CD31. A total of 98.6% of cells were positive for the established mesenchymal stem cell markers CD73 and CD90. In addition, 97.4% of cells were negative for CD34 and positive for CD105. These findings align with the joint IFATS and ISCT statement on ADSC markers [25].

**Figure 1:**
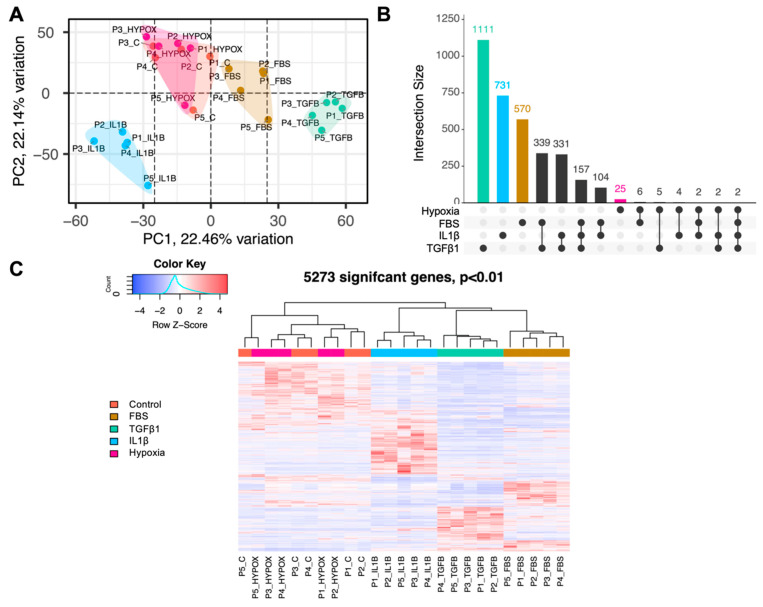
RNA-Seq analysis revealed distinct gene expression patterns between conditions. Coral colour = Control; Dark yellow colour = FBS; Turquoise colour = TGF-β1; Light blue colour = IL1-β; Pink colour = Hypoxia (**A**) Visualisation of principal component analysis (PCA) graphically shows the distance and, hence, differences between the conditions. (**B**) An intercept plot shows the number of uniquely expressed but also commonly expressed significant genes between conditions. The lines connecting the dots at the bottom imply which conditions are compared. (**C**) This heatmap is the result of unsupervised clustering and DESeq2 analysis. Row Z Scores are used for visualization. Genes with red are upregulated and blue downregulated.

Figure 1A shows that the different treatment groups cluster together after PCA analysis. However, there is a significant overlap between the control and the group exposed to hypoxia. This is confirmed by the upset plot with only 25 DEGs (Figure 1B) and the unsupervised clustering represented by a heatmap (Figure 1C). The FBS group was included in the experimental design to measure its effect on the transcriptome of ADSCs and to draw comparisons to other studies where media with 10% FBS is routinely used. FBS treatment is often utilised to simulate a wound-healing environment [33]. The FBS group elicits a similar response to TGF-β1 with the FBS-treated clustering between controls and TGF-β1 treated samples with an overlap of 339 DEGs (Figure 1A–C). There are 1111 DEGs for TGF-β1 and 731 DEGs for IL1-β. A total of 5273 DEGs were found during unsupervised clustering (Figure 1C).

### 3.2. The Most Highly Differentially Expressed ADSC Genes form Unique Clusters Depending on the Type of Treatment and Map to Selective Cellular Pathways

**Figure 2:**
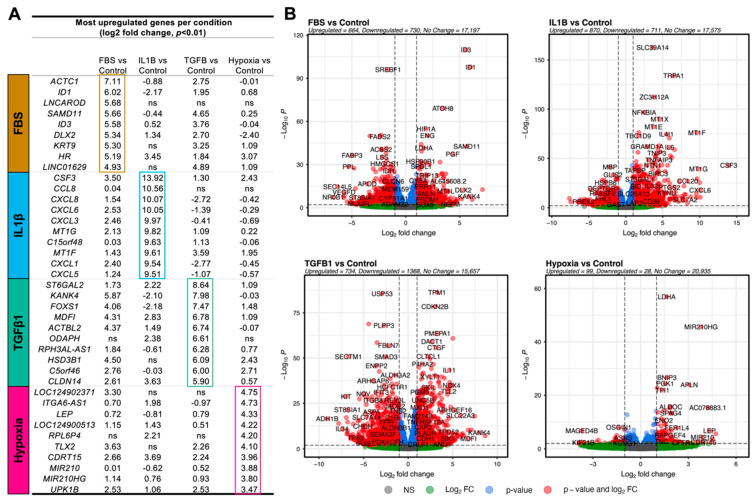
The top differentially expressed genes (DEGs) between conditions. (**A**) Shows the top 10 DEGs between conditions based on Log2 Foldchange (log2fc). (**B**) Volcano plots for each condition show the number of DEGs and several highly up- and downregulated genes. Red colour indicates genes are significantly (*p* < 0.01) and highly (log2fc ≤ 1 or log2fc ≥ 1) dysregulated. Green colour indicates they are highly dysregulated but not significantly. Blue indicates they are significantly but not highly dysregulated. Grey indicates they are neither significantly nor highly dysregulated.

We explored the DEGs more in-depth and selected the top 10 DEGs based on log2fc (Figure 2A). A variety of chemokines were highly upregulated in the IL1-β group including *CSF3*, *CCL8*, *CXCL8*, *CXCL6*, *CXCL3*, *CXCL1*, and *CXCL5*—all with a log2fc greater than 9. The corresponding Volcano plot shows that while there is a similar number of up- and downregulated genes, the upregulated genes tend to be more strongly and significantly upregulated (Figure 2B). The metallothioneins (MT) *MT1G* and *MT1F* were also amongst the most significantly upregulated. In the TGFβ1 group, the most significantly upregulated genes included *ST6GAL2*, *KANK4*, *FOXS1*, *MDFI*, and *CLDN14.* In addition, the profibrotic genes like *CTGF* (log2fc = 3.16), *ACTA2* (log2fc = 3.01), a variety of collagens including *COL1A1*, *COL4A1*, and *COL5A1*, and matrix metalloproteinases (MMPs) including *MMP10* and *MMP16* were upregulated. There were several disintegrins and metalloproteinases with thrombospondin motifs (ADAMTS) that were up- and downregulated. In Hypoxia, genes including *LEP*, *ITGA6*, *RPL6P4*, *TLX2*, and *CDRT15* were highly upregulated. Lower on the list but still significant were the proangiogenic factors *VEGFA* (log2fc = 1.11) and *ANGPTL4* (log2fc = 1.34). In the FBS group, *KANK4*, *ACTC1*, *ID1*, *ID3*, *KRT9*, and *DLX2* were most upregulated. The in vitro ADSC marker *ENG* was also upregulated (log2fc = 2.01)*. FGF2* was upregulated in all groups but Hypoxia. The full list of significant DEGs and their log2fc is available in Supplementary File S1.

### 3.3. Pathway Analysis Reveals Changes in the Biological Behaviour of ADSCs after Treatment

**Figure 3:**
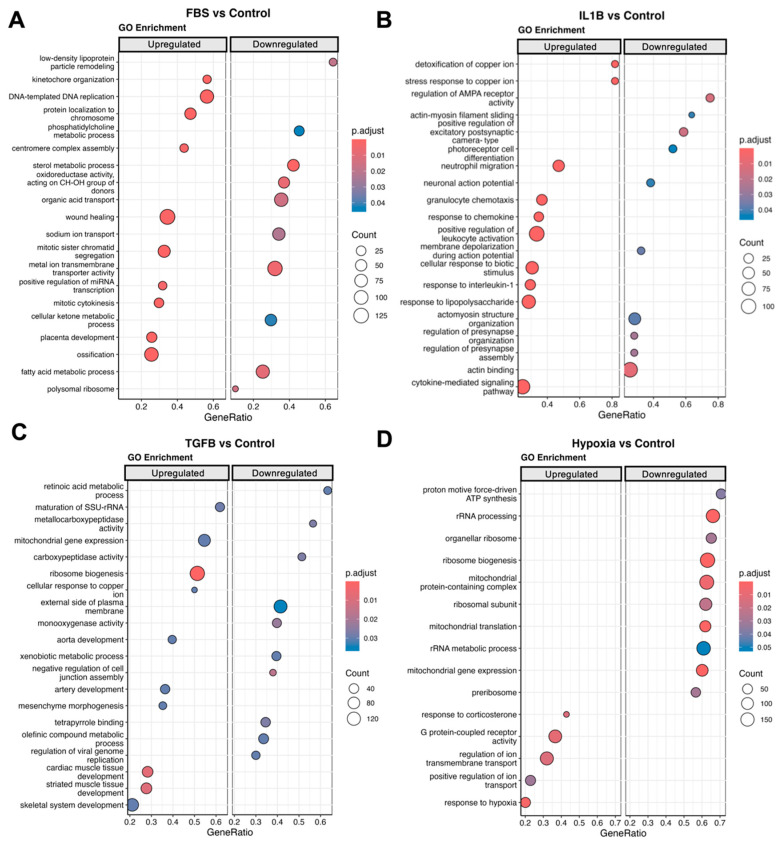
Gene set enrichment analysis (GSEA) of gene ontology (GO) pathways. (**A**) Demonstrates both up- and downregulated pathways between FBS and the control. (**B**) Demonstrates both up- and downregulated pathways between IL1-β and the control. (**C**) Demonstrates both up- and downregulated pathways between TGF-β1 and the control. (**D**) Demonstrates both up- and downregulated pathways between hypoxia and the control. Red colour indicates higher significance. Larger dot size indicates a higher count. Count is the number of genes that belong to a given gene set.

GSEA was performed and the results aligned with GO terms. Pathways associated with the cell cycle and replication were upregulated in the FBS group including centromere complex assembly, mitotic sister chromatid, and kinetochore organisation but also wound healing and ossification (Figure 3A). In the IL1-β group, immunomodulatory pathways were enriched including those for granulocyte chemotaxis, granulocyte migration, response to chemokine, response to interleukin-1, and cytokine production (Figure 3B), which are consistent with our results shown in Figure 2. Notably, various pathways associated with the cells’ synaptic excitatory potential and actin binding were downregulated. TGF-β1 treatment led to the upregulation of wound healing and skeletal system, cardiac, and striated muscle tissue development pathways (Figure 3C). Cells in hypoxia responded with the downregulation of mitochondria and cellular activity-associated pathways including mitochondrial gene expression, mitochondrial translation, ribosome biogenesis, and proton-motive force-driven ATP synthesis (Figure 3D). However, the response to hypoxia and the leptin-mediated signalling pathways were upregulated.

### 3.4. Genes of Selected Pathways Highlight the Association of ADSCs with Key Features Underlying Skin Fibrosis

**Figure 4:**
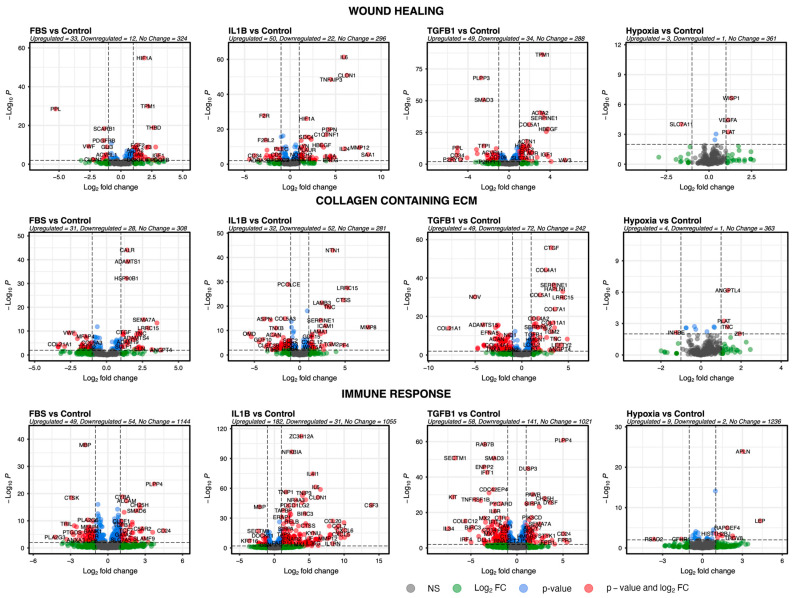
Wound healing, ECM-interacting, and immune response GO pathways. Volcano plots visualise the expression of genes belonging to pathways that have a possible effect on skin fibrosis. Red colour indicates genes are significantly (*p* < 0.01) and highly (log2fc ≤ 1 or log2fc ≥ 1) dysregulated. Green colour indicates they are highly dysregulated but not significantly. Blue indicates they are significantly but not highly dysregulated. Grey indicates they are neither significantly nor highly dysregulated.

Next, the GO pathways wound healing, immune response, and collagen-containing ECM were used to filter the results of DESeq2. These pathways were selected because of their possible implications for skin fibrosis. Wound healing contained a total of 425, collagen-containing extracellular matrix contained a total of 430, and immune response contained a total of 1903 genes. The results were visualised individually for each of the four comparisons to give a more focused insight into possible relevant effects for skin fibrosis (Figure 4).

We then compared the treatment groups by exploring which genes were commonly up- or downregulated between the conditions (Table 1). There were no commonly dysregulated genes from the selected pathways between all three conditions. Only when the log2fc threshold was lowered to 0.5, apelin (*APLN*) and vascular endothelial growth factor A (*VEGFA*) were both significantly upregulated.

Between IL1-β and TGF-β1 interleukins (*IL4R*, *IL21R*, *IL6*, *IL12A*, *IL1RAP*), basic fibroblasts growth factor (*FGF2*), ECM proteins (*COL7A1*, *COL10A1*, *TNC*), ECM degrading enzymes (*ADAMTS4*), immunomodulatory factors (*TGM2*, *LRCC15*, *RASGRP1*, *CD55*, *PDCD1LG2*, *SIRPA*, *PIK3AP1*, *PIK3CD*, *FCER1G*, *TNFRSF12A)*, haemostasis-associated factors (*SERPINE1*, *PLAUR*), oncogenes (*ABL1*, *CLCF1*), and a variety of other genes (*HIF1A*, *DUSP3*, *BTN2A1*, *PDE4D*, *PNP*, *STMP1*, *PXDN*, *EPG5*, *LRP8*, *CLCF1*, *ENDTP7*) were upregulated. *ST3GAL1*, *SRPX2*, and *CYR61* (*CCN1*) were also upregulated. There were also a variety of genes that were downregulated including ECM proteins (*COL5A3*, *COL21A1*, *ACAN*, *TNXB*, *ECM2*), ECM interacting factors (*ADAMTS15*, *ADAMTSL4*), and cell surface markers (*CD34*, *CD36*).

The preselected gene sets were used to filter the results of the DESeq2 analysis. Comparisons between all three conditions were based on a *p*-value smaller than 0.01 and a log2fc threshold greater than 0.5 or smaller than −0.5. For comparisons between TGF-β1 and IL1-β, the log2fc threshold was set to greater than 1 or smaller than −1.

## 4. Discussion

Skin fibrosis can result from chronic repetitive trauma, persistent inflammation, or exposure to toxins such as chemoradiotherapy. The tissue enters a cycle of ineffective wound healing with persistent activation of myofibroblasts and increased deposition of collagen, leading to elevated tissue stiffness [3,4,5]. Fat grafting into this chronic fibrotic tissue disrupts the tissue architecture and creates extra volume at the site of injection initially. A new wound is thus created with an influx of inflammatory cells and all other processes associated with tissue injury occur. However, it is also apparent that the long-term benefit, measurable at 6–12 months post-intervention, cannot be attributed solely to the created tissue volume since graft resorption rates up to 90% have been reported [34]. It is, therefore, highly likely that the graft has a long-lasting effect on changing tissue architecture at the macroscopic, microscopic, cell, and molecular levels.

ADSCs are widely believed to be responsible for the anti-fibrotic and anti-scarring effects of autologous fat grafts (AFGs). In vitro and in vivo research has demonstrated the immunomodulatory, regenerative, and proangiogenic effects of ADSCs as well as their ability to modulate the behaviour of fibroblasts—the key cell type in fibrosis [16,17,18,22,24]. However, these effects are currently being explained by only a few effector molecules including FGF2, HGF, IL6, IL10, IDO, PGE2, and VEGF [16,24]. Both in the initial phase of the application and the later stage of tissue remodelling, we hypothesise that ADSCs might be subject to TGF-β1, IL1-β, and hypoxia. Upon proinflammatory and profibrotic conditioning, transcriptomic analysis of ADSCs showed the upregulation of pathways including granulocyte chemotaxis, cytokine production, wound healing, and muscle tissue development. *FGF2*, *VEGFA*, *IL6*, various ECM components, and haemostasis-associated factors (*SERPINE1*, *PLAUR*) were commonly upregulated between the conditions.

The upregulated pathways (wound healing, chemotaxis) and commonly upregulated genes are all part of physiologic wound healing, causing haemostasis (*SERPINE1*, *PLAUR*), inflammation and immune cell influx (*IL6*), ECM proliferation (*COL7A1*, *COL10A1*, *TNC*), angiogenesis (*VEGFA*, *FGF2*), and tissue remodelling (*ADAMTS4*). Hence, the presence of ADSCs might enable the orchestration of wound healing, accelerating physiologic healing, and, hence, preventing the vicious cycle of ongoing inflammation. This potential mechanism of action appears relevant for the treatment of chronic diabetic ulcers where ulcers treated with ADSC-fibrin gels resulted in a greater and faster reduction in wound size and achieved complete healing in a significantly higher number of patients [35]. Notably, Forcheron et al. found an increased influx of lymphocytes in ADSC-grafted minipigs with radiation-induced fibrosis, with significantly improved wound healing [36]. This increased influx of immune cells might lead to a renewed immune response with acute rather than chronic inflammation and may contribute to the resolution of fibrosis possibly by clearing the fibrotic tissue of chronically activated fibroblasts or myofibroblasts. It is unknown how long ADSCs persist at the site of injection, although animal studies suggest they persist for up to 12 weeks and colocalise at the subcutis/dermis junction and in proximity to vasculature [36,37]. Angiogenesis also seems to be one major mechanism by which ADSCs could counteract fibrotic skin diseases such as radiation-induced fibrosis or scleroderma, where microangiopathy and tissue hypoxia are hallmarks [38,39,40,41]. Interestingly, studies have noted ADSCs to secrete proangiogenic factors including VEGFA [42], angiogenin, and FGF2 [43] after exposure to hypoxia [44,45,46], TGF-β1 [26], and TNF [47]. In this study, all the stimuli increased the expression of *VEGFA*, while *FGF2* was increased in both TGF-β1 and IL1-β. A significant mechanism described for the modulation of fibroblast activity by ADSCs involves the action of basic fibroblast growth factor (FGF2). Studies propose that FGF2 impedes the transition of fibroblasts into myofibroblasts and prompts apoptosis in myofibroblasts, consequently diminishing the synthesis of collagen [16]. FGF2 and HGF are believed to act together in an anti-fibrotic pathway [48]. While HGF is implicated in various anti-fibrotic effects of ADSCs [16], our recent research indicates a lack of high HGF expression by any cell type in the SVF [23]. In this study, *HGF* was significantly downregulated in both TGF-β1 and IL1-β. However, ADSCs may initiate *HGF* expression in response to fibroblasts during co-culture. Interestingly, *CD55* was upregulated significantly in various groups. CD55 or decay accelerating factor (DAF) acts as a protective factor against complement dysregulation, which is linked to the pathogenesis of idiopathic pulmonary fibrosis (IPF) [49].

However, overall, it is difficult to judge the anti-fibrotic effects of ADSCs after proinflammatory and profibrotic conditioning. The results are not unambiguous as it is possible to argue that some of the expressed and upregulated factors are profibrotic when not viewed in the context of acute wound healing. For example, IL6 is regularly described as one of the key drivers of wound healing and one of the anti-fibrotic mechanisms of ADSCs by promoting macrophage polarization [50]. But it also plays a role in the context of chronic inflammation including chronic inflammatory diseases, autoimmune diseases, and cancer and is increasingly regarded as a proinflammatory cytokine [51]. IL10, which is both anti-inflammatory and anti-fibrotic and is regularly described as a possible mechanism, was not significantly dysregulated in any of the groups [52]. The ADSCs’ regenerative capacity might also be affected by treatment, with both *CD34* and *CD36* being significantly downregulated. CD34 is associated with enhanced progenitor activity and stemness [53], while CD36 is a marker of adipogenesis [54].

The effect on the ECM is the most difficult to fully realise since it undergoes remodelling, consists of a variety of different proteins, and is the endpoint by which fibrosis is characterised. During wound healing, there is an interplay of ECM production (collagen, elastin, etc.) and degradation [8]. Matrix metalloproteinases (MMPs), their inhibitors (TIMPs), and ADAMTS are enzymes responsible for the remodelling and equilibrium/homeostasis of the ECM. When their balance is dysregulated due to repetitive trauma, chronic inflammation, or toxins, they can contribute to fibrosis [55]. Different subtypes of MMPs exist—paradoxically some with pro- and others with anti-fibrotic effects [56]. Here we found commonly upregulated (*COL7A1*, *COL10A1*, *TNC*) but also downregulated ECM components (*COL5A3*, *COL21A1*, *ACAN*, *TNXB*, *ECM2*). Similarly, ECM degrading enzymes were commonly up- (*ADAMTS4*) but also downregulated (*ADAMTS15*). Following exposure to TGF-β1, COL1A1 expression was upregulated, while exposure to IL1-β led to an increase in COL3A1 expression, both representing classical collagens that are upregulated in association with skin fibrosis [57]. Other genes that were upregulated and have an effect on the ECM were *ST3GAL1* (known to promote cell migration, invasion, and TGFB1-induced EMT [58]), *SRPX2* (known to promote cell proliferation and invasion [59]), and *CYR61* (*CCN1*) (known to promote EMT and is associated with fibrosis during skin expansion [60]). CCN1, however, also promotes collagen organization and causes angiogenesis [60].

A recent model explored the effect of adipose tissue-derived ECM (Ad-ECM) for treating radiation-induced skin fibrosis [61]. Ad-ECM treatment reduced proinflammatory and fibrotic gene expression and increased anti-inflammatory cytokines and bone marrow cell migration. Ad-ECM also increased *IL4*, *IL5*, and *IL15* expression, alleviating radiation-induced fibrosis. This underlines the assumption that secreted factors from ADSCs or cells in adipose tissue hold the key to understanding the positive effects of AFG. Even the Ad-ECM at baseline without stimuli and without graft inherent cells such as ADSCs seems to have promising alleviating effects, although this study does not elucidate which factors are responsible for this. In a recent in-depth examination of the secreted factors of ADSCs, researchers conducted a comprehensive review of proteomics databases [62]. This compared secretomic studies focusing on normoxic, hypoxic, or cytokine-exposed ADSCs, revealing only eight common proteins in normoxic secretomes (*COL1A1*, *COL1A2*, *COL3A1*, *COL6A1*, *COL6A2*, *FN1*, lumican, and osteonectin), no commonalities in hypoxic secretomes, and nine in cytokine-exposed secretomes (TNFRSF11B, OAF, IL-6, *COL5A1*, TSG-6, *CXCL2*, and *CCL2*), all showing the consistent presence of extracellular matrix-related pathways. Notably, even inflammation-associated proteins (IL-6 [63], *CXCL2* [64], and *CCL2* [65]) are associated with various ECM components and processes. Inflammation-associated genes including *IL6*, *CXCL2*, and *CCL2* were also upregulated in our study after IL1-β stimulation as well as *COL5A1* being upregulated after TGF-β1 conditioning. Although our study explored transcriptomics and not proteomics, our work aligns with the study of Pinheiro-Machado et al. and, again, strongly underlines the involvement of ADSCs in ECM remodelling pathways. However, only up to 10% of cells in the SVF of adipose tissue are ADSCs [66]. Hence, the importance of the secretion and production of ECM components is possibly less compared to their ability to modulate cell behaviour and orchestrate ECM homeostasis.

We further explored the effect of FBS on ADSCs. While all the other treatment groups were kept in media supplemented with 0.1% FBS, the FBS group was exposed to 10% FBS, which is commonly used in in vitro experiments. However, FBS had a significant effect on ADSC behaviour, with more than 1300 DEGs compared to the control, and elicited a similar response as TGF-β1. This is unsurprising since FBS contains a myriad of proteins, growth factors, cytokines, and hormones, including TGF-β [67]. The ADSC marker *ENG* (*CD105*) is only gained through cell culture and, seemingly, exposure to FBS [68]. *ENG* was significantly upregulated in this study. For future research, we suggest using low serum media to not modify the ADSC behaviour and also make the research more comparable, as there can be significant batch effects and variability between FBS products [69]. In general, standardization between studies on ADSC culture and experimental setup is needed to enable conclusions on the therapeutic value of ADSCs [62]. On a different note, the combination of prolonged hypoxia and 0.1% FBS media, on ADSCs, resulted in only a few DEGs compared to the control group, and pathways related to mitochondrial and cellular activity including mitochondrial gene expression, mitochondrial translation, and ribosome biogenesis were downregulated. Hypoxia still resulted in the upregulation of proangiogenic factors such as *VEGFA* and *ANGPTL4*; however, the fold change was small. Previous research either used higher FBS supplementation, higher oxygen levels, or shorter treatment durations [44,45,46,70]. However, a study examining different proteomic studies performed on hypoxia-treated ADSCs also found few differentially produced proteins compared to the control [62].

## 5. Conclusions

Our study demonstrates characteristic transcriptomic changes in ADSCs upon in vitro fibrotic and inflammatory conditioning, mimicking stimuli relevant to skin fibrosis. We identified distinct gene expression patterns under various stimuli, highlighting the impact of IL1-β, TGF-β1, hypoxia, and FBS on ADSCs. IL1-β induced immunomodulatory and proinflammatory pathways, while TGF-β1 upregulated wound healing-related pathways. Dysregulation of key genes, including *FGF2*, *VEGFA*, interleukins, and extracellular matrix proteins, was observed across the conditions. These genes could present potential targets for further research to develop effective anti-fibrotic therapies for skin fibrosis. The findings contribute to our understanding of the molecular responses of ADSCs to a fibrotic environment.

## Figures and Tables

**Table 1:** Comparison of commonly up- and downregulated genes between conditions based on preselected gene sets from the GO database.

Pathway	Genes
**Immune Reaction**	
Up- or downregulated in all 3 conditions	*APLN* (↑)
Upregulated in TGF-β1 and IL1-β	*ST3GAL1*, *STYK1*, *IL4R*, *ABL1*, *IL21R*, *DUSP3*, *BTN2A1*, *PDE4D*, *PXDN*, *IL6*, *CH25H*, *EPG5*, *PIK3AP1*, *LRP8*, *FCER1G*, *IL12A*, *PIK3CD*, *RASGRP1*, *CLCF1*, *IL1RAP*, *CD55*, *PDCD1LG2*, *ENTPD7*, *SIRPA*, *PNP*, *STMP1*
Downregulated in TGF-β1 and IL1-β	*SECTM1*, *DOCK11*, *RAB7B*, *MEF2C*, *CD36*, *JAG1*, *MAP2K6*, *TRIL*, *FES*, *ACKR4*, *CRIP1*
**ECM Organisation**	
Up- or downregulated in all 3 conditions	None
Upregulated in TGF-β1 and IL1-β	*TNC*, *SRPX2*, *SERPINE1*, *COL7A1*, *COL10A1*, *CYR61*, *ADAMTS4*, *LRRC15*, *TGM2*
Downregulated in TGF-β1 and IL1-β	*COL5A3*, *NOV*, *TNXB*, *OMD*, *ACAN*, *MMRN2*, *COL21A1*, *KAZALD1*, *OGN*, *S100A10*, *ADAMTS15*, *ADAMTSL4*, *SOD3*, *CLEC3B*, *S100A4*, *TMEFF2*, *SBSPON*, *ECM2*
**Wound Healing**	
Up- or downregulated in all 3 conditions	*VEGFA* (↑)
Upregulated in TGF-β1 and IL1-β	*TNFRSF12A*, *PLAUR*, *B4GALT1*, *HIF1A*, *SERPINE1*, *HBEGF*, *IL6*, *FGF2*, *CYR61*, *FCER1G*
Downregulated in TGF-β1 and IL1-β	*F2R*, *ACVRL1*, *F10*, *CD34*, *CD36*, *PPL*, *TMEFF2*, *ENTPD1*, *P2RY12*

## Data Availability

The data will be made available upon reasonable request.

## Supplementary Materials

The following supporting information can be downloaded at https://www.mdpi.com/article/10.3390/cells13080693/s1, Figure S1: Flow cytometric analysis of human adipose-derived stem cells (ADSCs). File S1: Differentially expressed genes (DEGs) of the different conditions.
