# Supplementary figures and images for: Exploring Anti-Fibrotic Effects of Adipose-Derived Stem Cells: Transcriptome Analysis upon Fibrotic, Inflammatory, and Hypoxic Conditioning
Source: Cells. 2024 Apr 17;13(8):693. doi: 10.3390/cells13080693 (PMC11049044; doi:10.3390/cells13080693)

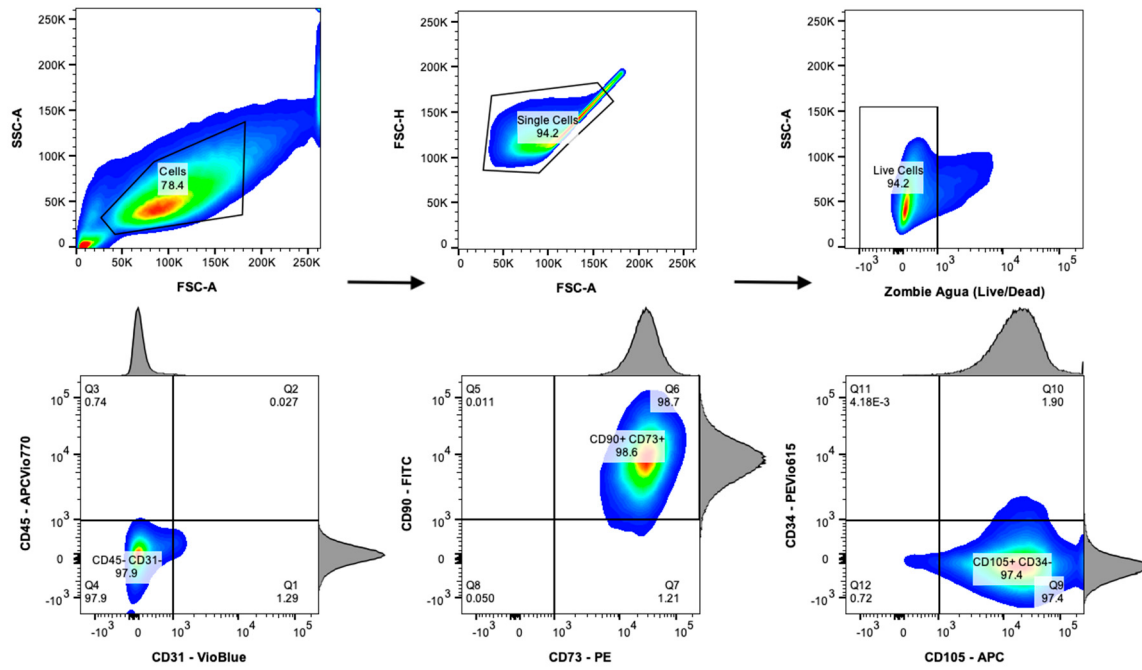

**Figure S1.** Flow cytometric analysis of human adipose-derived stem cells (ADSCs).

Supplement: Supplementary file 1 [file cells-13-00693-s001.zip › Figure S1.pdf]
